# Infectious complications and transplant outcomes in durable left ventricular assist device patients receiving immunosuppressive therapy: case series

**DOI:** 10.1093/ehjcr/ytag455

**Published:** 2026-06-25

**Authors:** Hirotaka Kawauchi, Takuma Sato, Satsuki Fukushima, Chisato Izumi, Yasumasa Tsukamoto

**Affiliations:** Department of Transplant Medicine, National Cerebral and Cardiovascular Center, 6-1 Kishibe Shin-machi, Suita, Osaka 564-8565, Japan; Department of Transplant Medicine, National Cerebral and Cardiovascular Center, 6-1 Kishibe Shin-machi, Suita, Osaka 564-8565, Japan; Department of Cardiovascular Surgery, National Cerebral and Cardiovascular Center, 6-1 Kishibe Shin-machi, Suita, Osaka 564-8565, Japan; Department of Heart Failure and Transplantation, National Cerebral and Cardiovascular Center, 6-1 Kishibe Shin-machi, Suita, Osaka 564-8565, Japan; Department of Transplant Medicine, National Cerebral and Cardiovascular Center, 6-1 Kishibe Shin-machi, Suita, Osaka 564-8565, Japan

**Keywords:** Left ventricular assist device (LVAD), Immunosuppressive therapy, LVAD-specific infection, Heart transplantation, Myocarditis, Cardiac sarcoidosis, Case report

## Abstract

**Background:**

The long-term prognosis of patients supported by durable left ventricular assist devices (LVAD) while receiving immunosuppressive therapy remains unclear. Underlying conditions such as cardiac sarcoidosis, recurrent myocarditis, or post-organ transplantation may necessitate concomitant immunosuppressive therapy during LVAD support. In particular, patients requiring prolonged immunosuppressive therapy exceeding low-dose corticosteroids represent a clinically challenging population in whom infectious complications and long-term outcomes have not been well characterized.

**Case summary:**

We retrospectively reviewed 241 consecutive LVAD recipients (HeartMate II and HeartMate 3) at a single centre (2013–24). Five patients (2.1%) required prolonged immunosuppressive therapy exceeding low-dose corticosteroids. Underlying cardiac conditions included myocarditis (*n* = 3), cardiac sarcoidosis (*n* = 1), and dilated cardiomyopathy following liver transplantation (*n* = 1). The mean duration of immunosuppressive therapy during LVAD support was 2.3 years (0.8–4.5 years). One patient developed LVAD-specific infections including driveline infection. Two underwent successful heart transplantation without perioperative complications. One was tapered to low-dose corticosteroid monotherapy due to a non-device-related infection, and two remained stable on LVAD support.

**Discussion:**

This case series underscores the complexity of managing LVAD patients requiring extended immunosuppressive therapy and highlights a unique and underreported patient population. Our findings suggest that successful heart transplantation may be achievable in immunosuppressed LVAD patients when supported by vigilant infection surveillance and individualized treatment strategies. This topic is of increasing clinical importance, particularly in regions where DT indications have recently been expanded.

Learning points

- Infections in immunosuppressed LVAD patients may follow an atypical course, requiring a high index of clinical suspicion and proactive self-management for early detection.
- In LVAD patients receiving prolonged immunosuppressive therapy, vigilant infection surveillance and individualized treatment strategies are warranted.
- Successful heart transplantation may remain achievable in immunosuppressed LVAD patients with careful management, although larger studies are needed to define the infectious risk in this population.

## Introduction

Durable left ventricular assist devices (LVAD) are increasingly utilized for the treatment of end-stage heart failure. In some regions, the annual number of heart transplantations (HT) remains limited due to a shortage of deceased donors. Most patients underwent LVAD implantation and waited an average of more than four years for a HT, necessitating extremely long-term LVAD management.^1,2^ This prolonged reliance on mechanical circulatory support underscores the importance of infection control and long-term management. Long-term LVAD therapy is always associated with the risk of infection, and the development of LVAD-specific infections is a major concern. In the United States, changes in allocation system have shifted LVAD use primarily towards destination therapy (DT).^3^ Similarly, DT has recently been introduced in other regions facing severe donor shortages, and the number of patients receiving LVAD implantation is increasing globally. These trends highlight the need to clarify the medium- to long-term prognosis of LVAD patients with various clinical backgrounds.

Underlying conditions such as cardiac sarcoidosis, recurrent myocarditis, or post-organ transplantation may require concomitant immunosuppressive therapy in LVAD patients. Although essential for disease control, immunosuppressive therapy has been reported to be associated with an increased risk of systemic and device-related infections. Immunosuppressive agents including corticosteroids, calcineurin inhibitors, antimetabolites, and mammalian target of rapamycin inhibitors, can impair host defence mechanisms, making patients more susceptible to bacterial, fungal, and opportunistic infections.^4^ Moreover, LVAD recipients inherently exhibit immune dysfunction. Prior studies have demonstrated altered T-cell responses, reduced antigen presentation, and heightened inflammatory cytokine activity in these patients—even in the absence of pharmacologic immunosuppression.^5–8^ The combination of LVAD-induced immune alterations and immunosuppressive drug exposure may therefore result in compounded infectious risk.

Despite these concerns, the long-term outcomes of LVAD patients receiving immunosuppressive therapy exceeding low-dose corticosteroids remain poorly described in the literature. To address this gap, we retrospectively reviewed 241 consecutive adult LVAD recipients (HeartMate II and HeartMate 3) at a single centre and identified five patients (2.1%) who received immunosuppressive therapy exceeding low-dose corticosteroids (defined as prednisolone ≥10 mg/day^9^ or use of any nonsteroidal immunosuppressant) for more than 3 months. We describe their clinical course, immunosuppressive strategy, infectious complications, and transplant outcomes, aiming to clarify issues and feasibility of managing this clinically challenging subgroup.

## Summary figure

**Figure:**
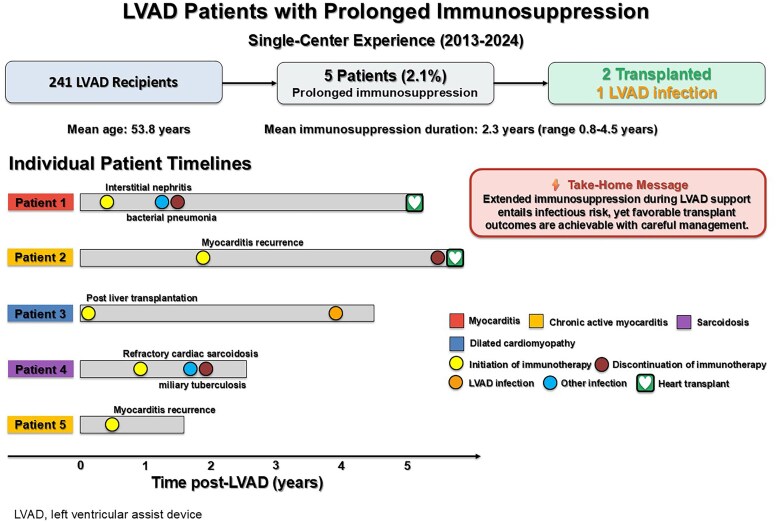


## Case presentation

Patient demographics, immunosuppression indications and regimens at LVAD implantation, and clinical outcomes and prophylactic antimicrobial use are summarized in *Tables 1–2*.

**Table 1:** Patient demographics and immunosuppression indications and regimen at LVAD implant

Patient no.	Sex	Age (years)	Primary cardiac disease	LVAD types	INTERMACS profile	Reasons for using immunosuppression	Before LVAD implantation	While on stable LVAD
1	M	48	myocarditis	HMⅡ	3	Interstitial nephritis	None	PSL 10–30 mg
2	F	42	myocarditis	HMⅡ	3	Chronic active myocarditis	PSL 5 mg	PSL 3 mg + CyA(80–100 ng/ml)
3	M	58	DCM	HM3	1	Liver transplantation	TAC, MMF	TAC + MMF + EVL ⇒ TAC
4	F	62	Sarcoidosis	HM3	3	Sarcoidosis	PSL 5 mg	PSL 5 mg + MTX4 mg ⇒ PSL 5 mg
5	F	59	myocarditis	HM3	2	Chronic active myocarditis	None	PSL 7.5 mg + CyA(100 ng/ml)

CyA, cyclosporine; DCM, dilated cardiomyopathy; EVL, everolimus; F, female; HM3, Heart Mate3; HMⅡ, Heart MateⅡ; LVAD, left ventricular assist device; M, male; MMF, mycophenolate mofetil; PSL, prednisolone; TAC, tacrolimus.

**Table 2:** Clinical outcomes and prophylactic antimicrobial use

Patient no.	Total LVAD support duration (years)	Duration of immunosuppressive therapy exceeding low-dose corticosteroids (years)	Heart Transplant	LVAD-specific infections	Non-LVAD infections	Prophylactic antimicrobials
1	5.3	1.0	(+)	None	Pneumonia	None
2	5.8	4.1	(+)	None	None	TMP/SMX
3	4.5	4.5 (ongoing)	(−)	Driveline infection	None	None
4	2.6	0.8	(−)	None	Miliary tuberculosis	TMP/SMX
5	1.6	1.1 (ongoing)	(−)	None	None	TMP/SMX

LVAD, left ventricular assist device; TMP/SMX, trimethoprim/sulfamethoxazole.

### Patient 1

A 48-year-old male with myocarditis presented with progressive heart failure and was classified as INTERMACS profile 3 at the time of evaluation. He underwent HeartMate II implantation as a bridge to transplant (BTT). Postoperatively, he developed interstitial nephritis and was treated with prednisolone at doses ranging from 10 to 30 mg/day, which were gradually tapered during follow-up. The duration of immunosuppressive therapy exceeding low-dose corticosteroids was 1.0 year. During the observation period, he was hospitalized with bacterial pneumonia, which resolved with intravenous antibiotic therapy. No LVAD-specific infections were observed. He subsequently underwent successful heart transplantation without any major perioperative complications and remained free from graft dysfunction and immunological rejection.

### Patient 2

A 42-year-old female with chronic active myocarditis was classified as INTERMACS profile 3 and underwent HeartMate II implantation as a BTT. Prior to LVAD implantation, she had been receiving prednisolone 5 mg. Following implantation, immunosuppressive therapy was discontinued; however, due to recurrence of myocarditis, prednisolone 3 mg and cyclosporine A (CyA) with a target trough level of 80–100 ng/ml were initiated. The duration of immunosuppressive therapy exceeding low-dose corticosteroids was 4.1 years. She did not experience any infectious complications during the observation period. She successfully underwent heart transplantation without perioperative complications and remained free from graft dysfunction and immunological rejection.

### Patient 3

A 58-year-old male with dilated cardiomyopathy (DCM) following liver transplantation presented with advanced heart failure and was classified as INTERMACS profile 1. He underwent HeartMate 3 implantation as a BTT. Due to his history of liver transplantation, he was managed with a triple-drug immunosuppressive regimen consisting of tacrolimus (TAC), mycophenolate mofetil, and everolimus. This regimen was subsequently simplified to TAC monotherapy due to stability in graft function and to reduce the risk of infections. The duration of immunosuppressive therapy exceeding low-dose corticosteroids was 4.5 years. During LVAD support, he developed a localized driveline infection that necessitated surgical debridement, long-term antibiotic treatment, and driveline relocation. At the time of analysis, he remained on the transplant waiting list with stable LVAD function and ongoing immunosuppressive therapy.

### Patient 4

A 62-year-old female with cardiac sarcoidosis was classified as INTERMACS profile 3 and underwent HeartMate 3 implantation as a bridge to candidacy. She had been receiving maintenance therapy with prednisolone 5 mg; however, due to refractory cardiac sarcoidosis, methotrexate (MTX) 4 mg was subsequently introduced. The duration of immunosuppressive therapy exceeding low-dose corticosteroids was 0.8 years. During the observation period, she developed miliary tuberculosis, leading to initiation of anti-tuberculosis treatment and discontinuation of MTX. Consequently, her immunosuppressive regimen was switched to corticosteroid monotherapy. No LVAD-specific infections were observed. At the time of analysis, she remained on the transplant waiting list with stable LVAD function.

### Patient 5

A 59-year-old female with chronic active myocarditis was classified as INTERMACS profile 2 and underwent HeartMate 3 implantation as a BTT. She was treated with prednisolone 7.5 mg and CyA with a target trough level of 100 ng/ml during LVAD support. The duration of immunosuppressive therapy exceeding low-dose corticosteroids was 1.1 years. She did not experience any infectious complications during the observation period. At the time of analysis, she remained on the transplant waiting list with stable LVAD function and ongoing immunosuppressive therapy.

## Discussion

This case series underscores the complexity of managing LVAD recipients requiring extended immunosuppressive therapy. Although small, this case series highlights a unique and underreported patient population requiring complex management. Although immunosuppressive therapy has been associated with both LVAD-specific and non-LVAD infections in prior reports, LVAD-specific infections can be particularly severe and therefore warrant close attention in LVAD patients. Notably, one of five patients experienced LVAD-specific infection. These findings underscore the urgent need for careful clinical decision-making, close infection monitoring, and tailored immunosuppressive strategies in such patients.

LVAD therapy is associated with a known baseline risk of infection due to the presence of a percutaneous driveline, long-term foreign-body exposure, and impaired lymphocyte function, making patients vulnerable to LVAD-specific infections. Moreover, patients who develop device-related infections have been reported to have worse prognosis after heart transplantation.^10–12^ Studies have shown that LVAD recipients demonstrate features of immune dysregulation even in the absence of pharmacological immunosuppression.^5–8^ When immunosuppressive agents are added—whether for sarcoidosis, myocarditis, or post-transplant indications—this immunological vulnerability may be further exacerbated. Previous studies have shown an increasing trend in LVAD-specific infections among LVAD recipients receiving immunosuppressive therapy, but the small number of cases leaves room for discussion.^13^

A key point of concern is that some infections may remain occult or manifest atypically under immunosuppression, delaying diagnosis and treatment. Even when LVAD-specific infections develop, wound culture tests often remain negative and inflammatory responses are frequently mild under immunosuppression. However, LVAD patients perform daily self-management such as driveline disinfection and are sensitive to subtle wound changes such as increased exudate or erythema. This high level of patient self-management may contribute to avoiding LVAD-specific infections that require hospitalization. This characteristic may be related to the fact that only one out of five cases in this case series developed LVAD-specific infections, despite concerns regarding infectious complications associated with immunosuppressant use.

The immunosuppressive regimens in our cases were diverse and adapted according to underlying conditions and tolerability. While corticosteroids were used in four cases, adjunctive agents varied, and tapering schedules were individualized. Importantly, most patients tolerated prolonged combination therapy, and two of them ultimately underwent successful HT. This suggests that with appropriate monitoring and antimicrobial prophylaxis, immunosuppression during LVAD support is feasible. However, except for two patients who required prolonged immunosuppressive therapy, the duration of higher-dose immunosuppression was relatively limited (0.8–1.1 years), although one of these patients continues therapy at the time of this report and the duration may increase over time. The difference in the duration of higher-dose immunosuppression among patients should be considered when interpreting the observed infection outcomes. Longer follow-up will be needed to determine whether patients with ongoing immunosuppressive therapy remain at similarly low risk over time. Furthermore, three of five patients received trimethoprim/sulfamethoxazole for Pneumocystis jirovecii prophylaxis during immunosuppressive therapy exceeding low-dose corticosteroids (*Table 2*). While this prophylaxis primarily targets non-LVAD-specific opportunistic infections, it may have contributed to reducing the overall infectious burden in our cases.

Our study also highlights the broader context of transplant access. In regions where waiting times are prolonged, immunosuppressed LVAD patients may remain on device support for years. During this period, even minor infections may compromise transplant eligibility or lead to delisting. Accordingly, aggressive infection control protocols—including driveline care, early antimicrobial intervention, and patient education—should be prioritized. Recently, LVAD patients receiving immunosuppressive therapy have also become eligible for DT indication in such regions, and the expected increase in such cases further emphasizes the clinical importance of this topic.

Current ESC Guidelines for Heart Failure (2021/2023) emphasize the importance of infection management in LVAD patients; however, specific recommendations for patients receiving concomitant immunosuppressive therapy are not provided. This case series provides evidence to address this guideline gap and highlights the importance of individualized management strategies.

From a research standpoint, our data reinforces the need for multicentre registries to systematically evaluate immunosuppressed LVAD recipients. Key outcomes such as infection-free survival, transplant rate, rejection incidence, and long-term mortality should be assessed prospectively. Additionally, the development of risk stratification tools specific to immunosuppressed LVAD patients could guide personalized therapy. Our findings suggest that successful transplantation may remain achievable in immunosuppressed LVAD patients when accompanied by rigorous infection control and individualized management, although larger, controlled studies are needed to clarify the infectious risk in this population.

This study has several limitations. First, it is a small, retrospective case series from a single centre. Second, the heterogeneity in immunosuppressive regimens limits direct comparisons. Third, the absence of a matched control group of LVAD patients without immunosuppressive therapy precludes definitive conclusions regarding the additive infectious risk of concomitant immunosuppression. For contextual comparison, a large registry study of 27 493 durable continuous-flow-LVAD patients reported a 5-year freedom from device-related infection of 61% for magnetically levitated devices.^14^ In our cases, one of five patients (20%) developed an LVAD-specific infection, but the small sample size and differences in follow-up duration preclude direct comparison with large registry data. Accordingly, our findings should be considered hypothesis-generating, and larger, prospective, controlled studies are needed to evaluate the infectious risk in immunosuppressed LVAD patients.

Despite these constraints, we believe the detailed clinical narratives provide meaningful insights for clinicians facing similar patient scenarios.

In conclusion, the incidence of LVAD-specific infections in LVAD patients receiving immunosuppressive therapy exceeding low-dose corticosteroids may not be as high as previously assumed. However, diligent infection control and individualized immunosuppressive strategies are believed to play a crucial role in achieving such favourable outcomes. Future multicentre collaboration is essential to develop risk-adapted protocols that ensure both safety and transplant eligibility for immunosuppressed LVAD recipients.

## Lead author biography


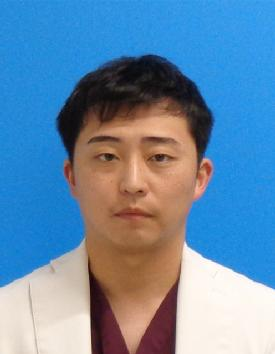
Hirotaka Kawauchi graduated with an MD degree in 2017 from the Kochi University School of Medicine, Japan. He is currently working at the Department of Transplant Medicine, National Cerebral and Cardiovascular Center (NCVC) in Osaka, Japan. His principal fields of interest are heart transplantation, mechanical circulatory support, and ventricular assist devices.

## Data Availability

The deidentified participant data will be shared upon reasonable request to the corresponding author for academic purposes. No additional documents will be available.
